# Validity of screening tools for emotional problems in school children

**DOI:** 10.4103/0019-5545.58896

**Published:** 2010

**Authors:** Shamshad Begum, K. Nagaraja Rao, C. Y. Sudarshan

**Affiliations:** Department of Psychiatry, JJM Medical College, Davangere - 577 004, Karnataka, India

**Keywords:** Screening instrument, school children, validity

## Abstract

**Background:**

Emotional problems in school children may result in low level of scholastic performance. The recognition of these disorders needs effective screening tools. The choice lies between self assessment tools or observation based tools. Majority of studies use screening tools based on parental or teachers' observation.

**Aim:**

This study was designed to compare a self-assessment based screening tool (general health questionnaire; GHQ) with a parental observation based screening tool (CPMS-Childhood Psychopathology Measurement Schedule).

**Materials and Methods:**

Two hundred and eighteen school children were selected through multistage random sampling. The study was conducted in three stages. In the first stage, all the students were administered six-item version of GHQ to screen for emotional problems. Raven's Progressive Matrices was administered to evaluate IQ. In the second stage, parents assessed their child's behavior using CPMS. In the third stage, all students were subjected for detailed clinical work-up.

**Statistical Analysis:**

Criterion validity of the tools used and their comparison.

**Result:**

GHQ had high sensitivity and specificity compared to CPMS in relation to clinical interview.

**Conclusion:**

It is found that GHQ is a better screening tool than CPMS in children aged between 13 and 14 years.

## INTRODUCTION

Emotional problems contribute to serious learning and health impairments in children. Indian studies report emotional problems in school children ranging from 10 to 74%.[1,2] However in a review article of school-based prevalence studies covering the period from 1972 to 2002, prevalence rates of 3.23 to 36.50%[3] were reported.

Assessment of emotional problems in children poses a challenge. Perception of non-psychotic emotional problems is usually individually determined. They are subjective in nature. However the onus of recognition of these disorders is invariably left to parents or teachers.

Emotionally disordered/disturbed children are under the care of parents. When parents do not recognize or not give much credence to the disturbance, there is no scope for intervention. This may result in devastating effect on growth of child's personality and learning process. The negative effect on learning process may result in low level of scholastic performance, though no overt psychiatric morbidity is recognizable in clinical sense.

Some studies have used GHQ screening tool and clinical interview in school children in the age range of 8 to 10 years[4] and 12 to 16 years.[5–8] However, in a review article covering school-based studies, none of the studies have compared any of self-report screening instruments with observation-based screening instruments.

This raises the question as to which is an efficient method to elicit emotional problems in children-self-assessment tools or observation-based tools?

Majority of studies use screening tools based on parental or teachers' observation to detect emotional disorders in school children.[3]

Thus it is worthwhile to compare self-assessment tools and tools relying on parental observation in assessing presence of emotional problems in children. Therefore the study was designed to compare a self-assessment based screening tool (GHQ) with a parental observation based screening tool (CPMS).

## MATERIALS AND METHODS

Students studying in tenth standard in an urban city formed the sample of the study. All the Kannada medium high schools situated in an urban city were stratified based on type of school (government, government aided, private), medium of instruction (English, Kannada and Urdu) and gender (girls, boys). Private schools were excluded as no private schools had schools exclusively for boys. One school each from government, government-aided, girls and boys schools were randomly selected for the study. Further, equal number of students were selected randomly from all. Totally there were 240 students. Twenty two students dropped out of the study for various reasons. Finally there were 218 students as the sample. To detect emotional problems in the sample, GHQ was administered to students, CPMS was administered to parents of the children. Tools used are: Six-item version of GHQ[9] and Child Psychopathology Measurement Schedule (CPMS).[10]

### The study was conducted in three stages

#### First stage: Initial screening of students

After obtaining information about socio-demographic details from parents and school records, all the students were administered six-item version of Goldberg's general health questionnaire (GHQ) to screen for emotional problems. Raven's Progressive Matrices was administered to evaluate IQ.

#### Second stage: Assessment by the parents

Parents were individually contacted and were requested to assess their child's behavior in the last one year and to fill the information in the Childhood Psychopathology Measurement Schedule (CPMS).

#### Third stage: Detailed clinical interview of students

Students who scored positive on any item on the GHQ and/or a score of 10 and above on CPMS were considered as cases. All subjects (cases and non-cases) were subjected for detailed clinical work-up by using a semi-structured interview. After detailed assessment, the psychiatric diagnoses were ascribed according to DSM-IIIR criteria.

## RESULTS

Out of 218 students, 131 were positive on GHQ and 98 students were positive on CPMS. Students who were positive on both scores were only 67. When these 168 students who were positive on GHQ and/or CPMS were subjected to detailed clinical assessment, 135 cases were clinically positive for emotional problems. In addition among the 50 cases, who were negative on screening instruments, 17 cases were positive on clinical interview [Table 1].

**Table 1:** Emotional problems in school children

Emotional disorder	GHQ N-218	CPMS N-218	Interview N-218
Present	131 (60)	98 (45)	152 (70)
Absent	87 (40)	120 (55)	66 (30)

Figures in parenthesis are percentages

**Figure 1:**
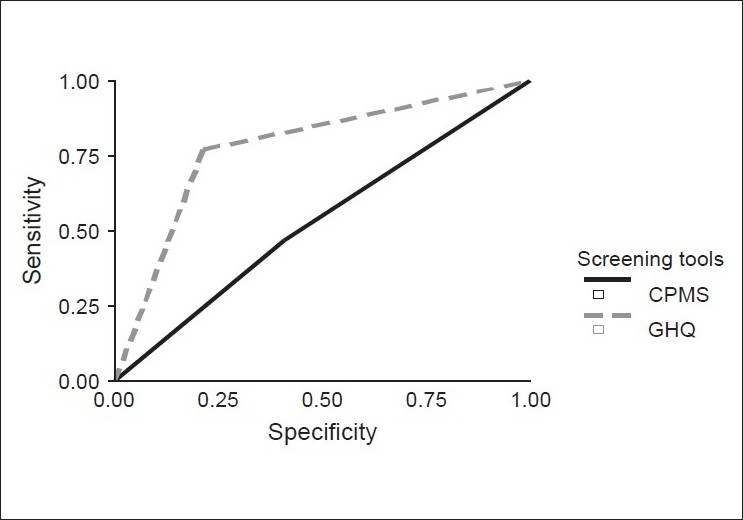
ROC curve of clinical interview vs GHQ and clinical interview vs CPMS

After clinical interview, out of 131 positive cases on GHQ, 117 were true positive cases and 14 were false positive cases. Out of the 87 negative cases, 35 were false negative cases. Thus sensitivity of GHQ screening tool was 0.77 and specificity was 0.79 with a false negative figure of 0.23 and false positive figure of 0.21. Positive Predictive Validity (PPV) was 0.89. The sensitivity and specificity of this instrument is almost equal. The Kappa value i.e. the agreement between the Clinical Interview and GHQ was 0.52, which was significant. After clinical interview, out of 98 positive cases on CPMS, 72 were true positive cases and 26 were false positive cases. Out of the 120 negative cases, 80 were false negative cases. The sensitivity of CPMS screening tool was 0.47 and specificity was 0.61 with a false negative figure of 0.52 and false positive figure of 0.39. Positive Predictive Validity was 0.73. Thus CPMS had better sensitivity than specificity. The Kappa value was 0.06. When GHQ was compared with CPMS, the sensitivity and specificity of GHQ was 0.51 and 0.64 with false negative and false positive figures of 0.49 and 0.36, respectively. Thus GHQ had higher sensitivity and specificity and PPV independently as well as in comparison with CPMS. The kappa value was 0.225 [Table 2]. The area covered under ROC curve for GHQ also was higher at 0.779 than CPMS in which area covered was 0.529 [Figure 1].

**Table 2:** Criterion validity of the screening tools in school children

	Interview vs GHQ	Interview vs CPMS	GHQ vs CPMS
Sensitivity	0.77	0.47	0.51
Specificity	0.79	0.61	0.64
False −ve	0.23	0.52	0.49
False +ve	0.21	0.39	0.36
PPV	0.89	0.73	0.68
Kappa	0.52	0.06	0.22

## DISCUSSION

Screening instruments are good tools for quick identification of emotional problems. CPMS are considered as a good screening instrument for children in the age group of 4 to 14 years. This is an observation-based tool. GHQ is a good screening tool for adults and it is self-assessment questionnaire. However, some published[5,6,8] and unpublished[4,7] studies have used GHQ for screening psychiatric morbidity in children. These studies have used long version of GHQ, which are time consuming. In this study, a short six-item version of GHQ has been used. There are no Indian studies, which have compared self-assessment screening tool and observation-based screening tools which this study has attempted.

This study suggests that GHQ is a better screening tool than CPMS in children aged between 13 and 14 years. This finding suggest the hypothesis that the emotional problems being individually determined and subjective in nature, self-assessment screening tool (GHQ) should be more useful than the observation-based screening tools like CPMS.

One of the reasons for such a result in this study could be that the age group of the sample was in higher band of the suggested age for CPMS, that is 14 years. Therefore it is worthwhile to find out the age at which self-assessment screening tools are better suited than observation-based screening tools in children.
